# Nasogastric-tube decompression facilitates the pocket-creation method of gastric endoscopic submucosal dissection.

**DOI:** 10.1055/a-2127-7583

**Published:** 2023-08-01

**Authors:** Hisashi Fukuda, Yoshikazu Hayashi, Yuka Kowazaki, Takaaki Morikawa, Alan Kawarai Lefor, Tetsurou Miwata, Sawako Fujikura

**Affiliations:** 1 Department of Gastroenterology, Jyoban Hospital, Tokiwa Foundation, Iwaki, Fukushima, Japan; 2 Department of Medicine, Division of Gastroenterology, Jichi Medical University, Shimotsuke, Japan; 3 Department of Surgery, Jichi Medical University, Shimotsuke, Japan

The pocket-creation method (PCM) 1 was developed as an effective strategy for endoscopic submucosal dissection (ESD), and its usefulness in gastric ESD has been reported 2 .

Aspirating gas from the stomach and collapsing the lumen facilitates the PCM because it helps to keep the submucosa thick and improves the controllability of the endoscope ( Fig. 1 ) 3 . However, it is not efficient to aspirate gas while placing the electric knife in the accessory channel. It is also time-consuming to remove the device just to aspirate gas.

**Fig. 1:**
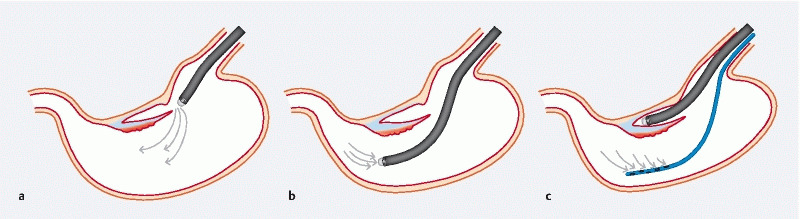
Schema of the stomach during gastric endoscopic submucosal dissection (ESD) using the pocket-creation method. **a** Gas insufflated into the pocket accumulates outside the pocket. **b** To aspirate unnecessary gas in the lumen, it is necessary to go out of the pocket. **c** When a nasogastric tube is placed, gas can drain spontaneously while the endoscope remains in the pocket.

We devised a method of gastric ESD using a nasogastric tube ( Video 1 ). A 14-Fr nasogastric tube was inserted through the nasal cavity and fixed at 65 cm ( Fig. 2 ). ESD with the PCM was then performed in the usual manner. During ESD, unnecessary gas and fluid drained through the tube without being aspirated through the endoscope ( Fig. 3 ). In addition, when bleeding occurred during the ESD, the blood also drained through the tube and no clots accumulated in the stomach. The tube placement did not cause any deterioration in endoscope controllability.

**Video 1** The pocket-creation method of gastric endoscopic submucosal dissection using a nasogastric tube.


**Fig. 2:**
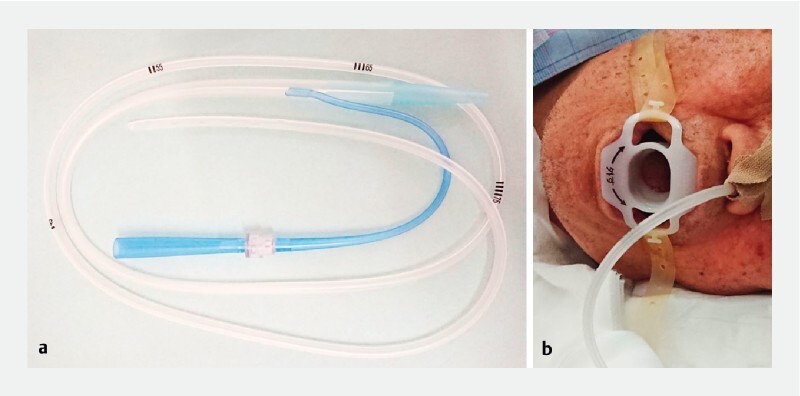
External pictures of the tube. **a** A 14-Fr nasogastric tube (Salem Sump; Cardinal Health, Dublin, Ohio, USA). **b** The nasogastric tube is inserted into the stomach and fixed at 65 cm.

**Fig. 3:**
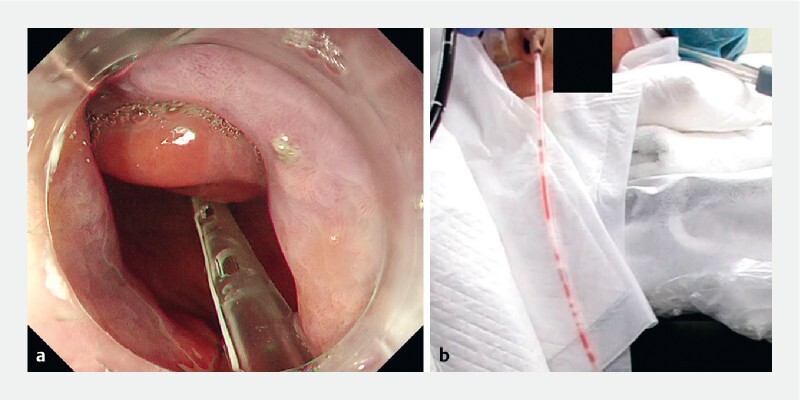
The nasogastric tube during ESD. **a** Endoscopic view of the nasogastric tube placed in the stomach. **b** Gas and fluid drain through the nasogastric tube.

This method has other advantages. Keeping the stomach at low pressure by removing gas seems to be less stressful for patients, which may allow sedation to be more effective. In addition, maintaining the stomach at low pressure may prevent Mallory–Weiss syndrome and vagal reflexes associated with hyperextension of the gastric wall. Even if an intraprocedural perforation occurs, leakage of air into the abdominal cavity can be minimized. In situations when the stomach is too collapsed to continue ESD, this can be solved by just clamping the tube with Pean forceps.

In conclusion, the nasogastric tube decompression technique facilitates gastric ESD using the PCM.

Endoscopy_UCTN_Code_TTT_1AO_2AG

## Notes

Endoscopy E-Videos

https://eref.thieme.de/e-videos
*E-Videos* is an open access online section of the journal *Endoscopy* , reporting on interesting cases and new techniques in gastroenterological endoscopy. All papers include a high-quality video and are published with a Creative Commons CC-BY license. Endoscopy E-Videos qualify for HINARI discounts and waivers and eligibility is automatically checked during the submission process. We grant 100% waivers to articles whose corresponding authors are based in Group A countries and 50% waivers to those who are based in Group B countries as classified by Research4Life (see: https://www.research4life.org/access/eligibility/ ). This section has its own submission website at https://mc.manuscriptcentral.com/e-videos
